# A Dissertation on the Pathology of the Bones, with Illustrative Cases; among Others, a Case of the Removal of Carious Ribs

**Published:** 1828-05

**Authors:** William A. M'Dowell

**Affiliations:** Fincastle, Virginia.


### PATHOLOGY OF THE BONES.

*A Dissertation on the Pathology of the Bones, with illustrative Cases; among others, a Case of the Removal of carious Ribs.* By WILLIAM A. M'DOWELL, M.D. of Fincastle, Virginia. (From the *American Medical Recorder.*)

THERE are few subjects connected with surgery, or I believe I might say with the healing art, of the same importance, that have been so much neglected as the pathology of the bones. Diseases of the bones are the most frequent in surgery, they are the most tedious, the most insidious, and the most loathsome: such considerations, we would suppose, should render a correct idea of their pathology matter of primary importance. But the fact is otherwise: though various diseases of those important supports are of daily occurrence to the surgeon, his treatment generally consists in little else than naming them, and, when christened, they are committed to the management of nature; or, on many occasions, by external emollient applications, relaxation or disease of the investing soft parts is added to the original malady.

The frequent occurrence of diseased bones early attracted my attention to their management: on consulting a variety of works with a view to elicit information, I met with such contrariety of opinions with regard to the causes, progress, and treatment of their diseases, that I was left unrequited and bewildered.

There exists so great a diversity even as to names and the distinctions of such diseases, that the practitioner is at a loss what name to give such cases as occur to himself; nor can he, in a great majority of them, adopt any appellation that will not be in opposition to some high authority; but his comfort may be, that he is countenanced by others equally imposing.

Only in one thing can any general accordance be discovered, which is to leave diseases of the bones, of whatever denomination, almost exclusively to the efforts of nature; which sometimes, after a tedious interval of loathsome and occasionally painful endurance, results in restoration; but more frequently in the necessity of amputating a limb, or in death. Very comfortable

The unsatisfactory result of my researches, arising from the contrariety of principles and the inefficacy of treatment on any authorised plan, induced me to investigate for myself; which investigation determined me in the adoption of pathological principles, and a mode of treatment, which I believe to be, for the most part, peculiarly my own.

It is with reluctance, and not without great diffidence of my ability to illustrate my positions, that I array myself in opposition to the doctrines of the respected and highly authoritative names of Bell, Boyer, and others; but the higher the authority by which error is propagated, the more extensive will be the injury we may expect it to produce, and the more imperative is the duty to expose it on detection.

Urged by such considerations, I am induced to submit to the profession the principles by which my practice is governed in the management of diseased bones, with several interesting illustrative cases. The plan of treatment alluded to is founded on the belief that the vis vitæ of the bones is generally inadequate, unassisted, to relieve them from diseases that have destroyed any portion of their structure; and that the interference and co-operation of the surgeon is more necessary in these diseases than almost any others that occur.

Besides the results of my own experience, many cases might be cited from the most eminent authorities in support of my views; particularly Mr. Hey's operations on the bones of the leg, for a disease which would be denominated, according to the nosology of Baron Boyer, "internal necrosis," and according to Messrs. John Bell and Samuel Cooper, "spina ventosa," but which Mr. Hey, perhaps with a view to avoid collision with any of those authorities, has introduced as a new disease, which he calls "abscess of the tibia." But none of these cases seem to have led their reporters to any more correct general conclusions; they still retained their preconceptions that bones possessed the capacity of separating mortified portions, after the manner and with the facility of muscular parts, without duly regarding their difference of structure.

Until such error in general principles is corrected, no extensive benefit can be derived from particular cases; for cases, whilst the surgeon yet labours under erroneous pathological views, constitute a guide to him only on the recurrence of a disease, a fac simile of the case successfully treated: cases are but facts, and special facts are so limited, that, to render them of extensive benefit to science, they must be constituted data on which to establish correct general principles.

Well satisfied that the pathological views of our most celebrated authors on diseases of the bones are incorrect, I shall, in the succeeding pages, from such facts as have occurred to myself, and from such as I have gleaned from others, endeavour to demonstrate such general methods of treating those diseases as seems to me to constitute an improvement in this branch of surgery.

In my detail of cases, some of which possess no novelty but the pathological views on which they were conducted, and which they are supposed to substantiate, as much brevity as consists with a correct idea of the situation of the patient and the nature of the case has been studied.

*Physiology and pathology of the bones.*—For the particular physiology of the bones, the reader is referred to works on that subject: it is sufficient here to observe, that all the bones have, like the soft parts, their arteries, veins, absorbents, and nerves. The blood-vessels destined for the nourishment of the external lamellæ are supplied principally from the periosteum; those destined for their interior structure generally enter near the middle of the long bones, and are distributed up and down in minute ramifications, throughout their cancelli or diploe.

The bones are endued with so little feeling that they are almost insensible to impressions, even of burning or decay, except when under some extraordinary excitement,* as from cold, &c. as is familiarly exemplified in carious teeth. Hence a bone may sometimes be utterly destroyed before it is discovered to be diseased. In the succeeding observations, to keep clear of ill-defined distinctions, I shall use the term necrosis for dead bone, from whatever cause arising; distinguishing affections of the lamellæ from those of the cancelli by the terms external and internal; conceiving, with Mr. Cooper and other good authority, that caries is of the same character with necrosis, and that it is necrosis of a smaller portion of a bone, (and that its extension throughout the entire bone constitutes complete necrosis,) that term will imply so much when hereafter used.

Necrosis proceeds from various causes, constitutional and casual. When arising from casualty, it generally proceeds either from denudation of the bone, from external violence, or from long continued application of cold, interrupting the internal structure. When the periosteum is by violence detached from the bone it invests, a clot of effused blood is interposed, which, if not speedily absorbed, (which it generally is,) presently brings on necrosis or caries of that part of the bone on which it lies, by ***cutting off its requisite supply of blood.*** In this, let it be understood, I conceive a marked difference to exist between the pathology of the bones and of

* John Bell says, "no pains are equal to those of the bones and joints;" but in his Principles of Surgery he states one case, which, on good data, he concludes originated in an injury inflicted on the bone ten years before disease was manifested; during all which time it had progressed with little or no pain. On dissection, it was found much bone had been destroyed. He also reports several other cases, less remarkable, but to the same purport.

the soft parts. The interruption of one or more of the smaller vessels conveying blood to soft parts, or even of very large vessels, is easily and speedily amended by dilatation of inosculating vessels: but in the bones, though their vessels too have their inosculations, yet those inosculating vessels cannot dilate with the same facility, nor to such extent, owing to the unyielding calibre of the long canals, through which they are transmitted.

Baron Boyer supposes necrosis from violence arises only from blows, or from pressure of sufficient force to depress some of the lamellæ, or otherwise directly injuring the tissue of the bone: but Mr. John Bell states many well-authenticated cases of necrosis, from violence so slight as not to interrupt continuity of the soft parts; some cases even arising from forcibly pulling the hair of the head, in which cases we cannot conceive the cause adequate to have produced the effect in any other way than by interrupting the necessary supply of blood to the bone beneath.

A clot of blood, remaining a length of time, produces necrosis of a scale of the lamellæ on which it lay, which excites the surrounding structure to the formation of pus, as a medium of separation between the living and the dead bone; or the clot of itself, producing irritation foreign to the nature of the part, may excite to the formation of pus. This fluid accumulating, either bursts through the periosteum and integuments, and is discharged, or encountering a firm resistance from the periosteum, (which is often the case,) attains the necessary space for its increased bulk, by extending the separation between the bone and its periosteum, sometimes entirely denuding it, and thus effecting its complete necrosis. This is what I would term external necrosis, and, unless the pus is evacuated and allowed a free vent, it becomes the cause of the extension of the disease from bone to bone, until sometimes many bones are affected, as in the following case:

I visited James Summerfield, of Newcastle, aged twenty-three, in autumn 1822, afflicted with what he termed white swelling. He believed the disease to have originated from a scuffle, in which his thigh had been hurt, two years before. In addition to a very small sinus, four or five inches above the knee, an abscess was pointing in the groin, which I opened. After evacuating the pus, injected fluids readily passed from one opening to the other; the whole femoris appeared to be denuded. This was the first case of the kind of importance that had occurred in my practice; conceiving it a hopeless one, I ordered the best authorised treatment. He lived upwards of two years afterwards.

No callus was deposited, nor was there any tendency to amendment discovered during that time. Before he died, suppuration had extended to the foot, and a sinus had formed over the posterior spinal process of the os ilium.

As external necrosis is of most frequent occurrence, and consequently most important to be well understood, we will first take a view of the changes the appearance of the bone undergoes, and the requisite treatment adapted to the changes of this particular form of the disease.

"The colour of a living bone," says Fallopius, "is white, delicately tinged with red; that of a dead bone, unmixed white; that of a putrid or carious bone, livid or black." Living bone is also distinguished from dead by freely bleeding when scraped, and, where only a small plate of lamellæ is carious, the sound bone around the plate is slightly elevated or swelled, and bleeds particularly free; easily marking the line of distinction.

When, from denudation, the external lamellæ of a bone is found necrosing, and we have reason to believe its internal structure is sound, we should use every effort to reanimate, or resuscitate (if I may use the phrase,) the necrosing portion.

1st. By making a free incision through the integuments at the most depending part to which pus has attained, and another at the upper, or at that part on which the injury was inflicted, with a view to discharge the pus, and stop the extension of the disease.

2d. By exciting the exterior of the bone and the interior of the periosteum, by stimulating washes; by passing setons, when the parts will admit it, from a superior to an inferior opening, along the bone; and by compression from without with a roller, or with the laced stocking.

The beneficial effects of this plan were strikingly exhibited in the following case:

Tom, a negro boy, the property of Mrs. Rowland, aged ten years, jumping from the top of a fence, fell, and struck the tibia against a rail that lay on the ground. A small tumor arose, which at the time was but little regarded. About fourteen days afterwards, general swelling of the leg commenced, attended with severe pain: the swelling soon became most where the original injury was inflicted, at which point his attending physician, Dr. S. O. Caruthers, made a small opening with a lancet, from which there was a free discharge of pus. But the swelling below still extended towards the foot, and he daily grew worse. Hectic fever supervened, with colliquative sweats and diarrhœa. Those symptoms were combated with the ordinary remedies: nevertheless, his danger became so eminent, that I was called in to amputate the leg, and visited him with that view on the 22d November, 1825. On enlarging the small issue already made between the tibia and tibialis anticus, to a size that admitted the introduction of the finger, both the tibia and fibula were found denuded as far as they could be examined, and appeared dead, exhibiting the white graveyard appearance. In conference with Dr. C., on representing the success of my restorative endeavours in cases I conceived to have been of similar character, we concluded to delay amputation, and make a trial of the kind. With such views, a large knitting needle (having no probe of sufficient length,) was introduced at the opening above named, and passed down along the naked tibia, passing between the two bones, and was cut out posterior to them, between the malleolus externus and the tendo Achillis; believing this to be the most depending situation to which pus had arrived. A stout skein of thread was passed from one opening to the other, and the roller was applied from the toes to the knee. I never visited him afterwards, but was informed by Dr. C. that the fever and constitutional symptoms, after this operation, quickly yielded to the same remedies they had baffled before, and that his recovery was rapid and complete.

By those means much may be done, if resorted to in time; nor should such treatment be omitted within any given time, unless the bone were found livid or black; in which event, the dead bone should be removed, or the limb amputated: but the former I should generally prefer, *even if it were the whole femoris*, for then nothing short of amputation at the hip could be substituted. Here my conclusions may be deemed hasty, inasmuch as the fact is well established that such dead bones have actually been found inclosed in newly generated callus. Such pathological information I deem serviceable only as affording the assurance that a speedy renewal of displaced bone, in a young subject of good constitution, may confidently be expected if unimpeded, which should admonish the surgeon that, in aid of nature, he should endeavour to remove all obstacles and impediments to her process.

Nowhere have I ever met with the history of the life, or cause of the death, of any one in whom such phenomena as inclosed bones have been discovered: hence my conclusion is, that those bones probably introduced their natural proprietors into the dissecting room. If so, the fact affords but an indifferent reason for the surgeon leaving them unmolested.

Case.—Virginia Harvey was, in the month of February 1816, at two years of age, through carelessness of her nurse, in her mother's absence, very much exposed to cold: the succeeding day she was unable to walk, complained very much of pain in the right leg, which was at first unattended with swelling or discoloration; but in about three weeks it swelled, pointed, and was opened about midleg, and pus was discharged, with some relief from pain, but with no other amendment. About six months after this, hectic symptoms becoming urgent, a consultation of physicians was called: an incision was made, exposing the bone from within about an inch of the head of the tibia, to within the same distance of the ankle-joint. The bone was denuded and black. The free evacuation of pus from this incision mitigated the hectic, and for a time relieved constitutional symptoms; but this black bone, dead and detached as it was from its periosteum, though as foreign to the surrounding parts as is the dead to the living, was, in conformity with the precepts of the best surgical authorities, left in its place, where it remained nearly six months longer, in which time it exfoliated, plate by plate, near the ankle, until the lower end was released, and projected through the integuments. In this situation it remained some time longer, and was cautiously moved a little every day by her attending physician. During all this time deep-seated abscesses, some of them very large, one after another, arose on different parts of her body, inducing the apprehension that the bones generally were diseased. Her physician at last discovered that callus was forming behind this black bone, and that it was giving the new bone a misshape, pulled it out, and found that an entire new bone had formed behind it, which was much bent backwards a little below the knee, from pressure by the upper head of the old tibia. Thirteen days after the removal of this dead bone, the leg had completely healed, together with every abscess upon her body; and, by the entire relief from hectic, which was removed with the bone, her strength had so much increased, that the little creature could not be prevented walking often around the room, aiding and supporting herself by holding to the furniture, which premature pressure increased the bend the new bone had already suffered, and caused a shortening of the leg of an inch and a half or two inches: the leg is, in other respects, equal to the other one; her constitution and general health, from that to the present time, has appeared as good as common.

The above case is given as related to me by the attending physician, Dr. Patterson, and by Miss Harvey's mother from memory; no notes having been taken of her case at the time of attendance. Dr. P. does not recollect with sufficient distinctness to determine whether this was a case of external or internal necrosis.

If the exterior of a diseased bone is discovered to be black only in parts, every effort should be made to reanimate so much of it as is not too decidedly carious, even though the whole bone be denuded. If in any encouraging degree successful, so soon as the lines of distinction could be designated, the carious portions should be forcibly detached, without wasting any time awaiting the dubious process of exfoliation; for, if exfoliation is desirable and useful, it is as useful if effected as if awaited by the surgeon.

Case.—Matilda, a mulatto woman, the property of General J. Preston, aged twenty-five, was visited 26th December, 1823: she had a deep-seated swelling, and pain below the middle posterior part of the thigh, which had been of several weeks' duration. Discutient liniments, blisters, mercurial plasters, and poultices, were applied without effect. On the 2d January, 1824, a free incision was made, and about a pint of pus discharged from a situation much deeper than external examination had led me to expect: the bone was denuded, as far as I could examine it up and down, to the extent of eight or nine inches; the opening was sustained by tents, and a roller was applied from the toes to the hip; at each dressing, tincture of myrrh was freely injected. By the 1st of March, amendment was such that only a small denudation of bone remained, not exceeding an inch. On the 2d of April, a carious plate was forcibly detached; eight or ten days afterwards, on examination, the bone could not be reached with the probe; and, on the 18th April, she was discharged cured.

No satisfactory account could be given of the origin of disease in this case.

The following case is introduced to exemplify the extreme tardiness and uncertainty of nature's process to exfoliate necrosing bone.

Bill, a negro man, the property of Mr. S. Kennedy, suffered a compound fracture of the femoris when a boy, from the falling of a tree upon him. Reunion of the bone was tedious, and, after it was completed, the opening through the integuments continued, and became fistulous. On the 9th June, 1821, I was called to examine him, (eight years after he had received the fracture.) The issue was situated about six inches below the trochanter major, through which the probe readily reached rough naked bone; the muscles of the thigh and leg of the diseased limb were flaccid and shrivelled, and the limb was but little more than half the size of the other. His general health was such that he could endure no fatigue, and caused him to pass most of his time in a recumbent posture.

By the advice and with the co-operation of Drs. Patterson and Madison, such an incision was made as admitted free examination of the bone. A caries was discovered about an inch in diameter, rough and black; it was surrounded and tightly embraced by the edges of the sound bone, which seemed disposed to close over it, resembling the appearance sometimes remarked in a tree, the recent growths of which are in the act of closing over a canker it has sustained from partial decortication, or from the removal of a chip.

This black scale, more than a quarter of an inch in thickness, and still very firm, was broken up from its place, and removed. The sinus healed in a few weeks; the boy regained his general health; and the diseased limb, in the course of a few months, recovered the healthful appearance and the full size of its fellow.

I am well satisfied, from observation and research, that carious lamellæ, left to nature, are more prone to reduplicate than to exfoliate. The very efforts of nature for self-preservation in those cases, when inadequate to the purpose, expedites the devastation; for bones are not so prone to decomposition through mere loss of vitality.

They moulder in burial-grounds for centuries without assuming the black colour they so very soon acquire in necrosis, Their inert durability has rendered them the only memorials of generations of men and animals swept from the face of the earth time out of mind.

To what, then, are we to ascribe their rapid decomposition in necrosis? To nought, I conceive, but the abortive efforts of nature's process in the adjacent sound structure to effect a release from an unnatural connexion with the dead. The inflammation and softening, preparatory to the formation of pus as a medium of separation, expedites the extension of necrosis; those softened and inflamed parts, irritated until exhausted by the protracted contact of an immovable caries, and enveloped in eroding pus,* grown acrid from age, are at last found necrosing in their turn. Hence the difficulty of arresting the progress of caries. If the process of exfoliation is depended upon, by the time the first scale is exfoliated, that part of the bone from which it has detached has been destroyed by its own over-action and over-excitement, and constitutes another subject for the same process.

The fact is familiar that, if a tooth becomes affected with a carious speck, if the caries is uninterrupted, it extends to the

* Whether erosion is ever produced by pus, I am aware is a controverted question; but, bland and emollient as it is on its recent formation, I was satisfied, by the following case, that it sometimes becomes acrid or eroding on confinement after its formation.

John Douglas, Esq. near Pattonsburg, struck a man in the mouth, and cut his knuckle on a tooth down to the bone: the cut healed, but soon afterwards the finger swelled, and, through mismanagement of a woman famed for curing swellings, it in a few months extended to the elbow, and the arm had to be amputated. I was invited by Dr. Patterson to assist him in the amputation. Examination after its removal discovered the anterior muscles of the forearm to have been completely destroyed: the skin contained the pus like a sac; the radius and ulna were entirely denuded, and of the dead white colour; the bones of the originally injured finger were livid.

I suppose those muscles to have been acted upon by the pus, for I cannot well imagine that the absorbents retained vital power enough to have effected their removal, when the organization of all the rest of the structure of the arm was destroyed.

destruction of the entire tooth; and we are also familiar with the fact that, if the speck is removed, the further progress of decay is arrested; but this is all so familiar that we forget a tooth is bone.

I have myself seen the caries of a tooth extended to the alveolar process.

In November, 1826, I saw a negro woman, the property of Mr. J. Thompson, who had a fistulous opening from the lower jaw, opposite the root of the largest grinder. Finding this tooth decayed, I extracted it: a carious portion of the alveolar process adhered to its outer root, to the extremity of which the tooth was decayed. The probe now passed through the fistulous opening into the mouth.

This opening had been of more than twelve months' standing, but healed in a few weeks after the extraction of the tooth.

[To be continued.]

---

